# Consumption of Antibacterials for Systemic Use in Slovakia: A National Study and the Quality Indicators for Outpatient Antibiotic Use

**DOI:** 10.3390/antibiotics10101180

**Published:** 2021-09-28

**Authors:** Tomas Tesar, Lucia Masarykova, Lubica Lehocka, Slavka Porubcova, Monika Cicova, Martin Wawruch

**Affiliations:** 1 Department of Organisation and Management in Pharmacy, Faculty of Pharmacy, Comenius University in Bratislava, 832 32 Bratislava, Slovakia; masarykova@fpharm.uniba.sk (L.M.); lehocka@fpharm.uniba.sk (L.L.); slavka.porubcova@nusch.sk (S.P.); cicova10@uniba.sk (M.C.); 2 Institute of Pharmacology and Clinical Pharmacology, Faculty of Medicine, Comenius University in Bratislava, 813 72 Bratislava, Slovakia; martin.wawruch@fmed.uniba.sk

**Keywords:** antibiotic consumption (AMC), defined daily dose (DDD)/1000 patient-days, quality indicators, anatomical therapeutic chemical classification system (ATC)

## Abstract

This paper aims to analyse the consumption of antibiotics in the Slovak health care system from 2011 to 2020. The data source on the consumption of antibiotics is sales data from SUKL and NCZI. The study employed the ATC/DDD Index and focused on the consumption of antibiotics in the primary care sector. Total antibiotic consumption decreased from 19.21 DID in 2011 to 13.16 DID in 2020. Consumption of beta-lactamase-sensitive penicillins, expressed as a percentage of the total consumption of antibiotics, decreased from 8.4% in 2011 to 4.2% in 2020. Consumption of the combination of penicillins, including beta-lactamase inhibitor, expressed as a percentage of the total consumption of antibiotics, increased from 16.2% in 2011 to 17.9% in 2020. Consumption of third- and fourth-generation cephalosporins, expressed as the percentage of the total consumption of antibiotics, increased from 2.0% in 2011 to 4.6% in 2020. Consumption of fluoroquinolones, expressed as the percentage of the total consumption of antibiotics, decreased from 10.7% in 2011 to 8.6% in 2020. Overall, antibiotic consumption significantly changed in Slovakia from 2011 to 2020. The ratio of the consumption of broad-spectrum to the consumption of narrow-spectrum penicillins, cephalosporins and macrolides decreased from 14.98 in 2011 to 13.38 in 2020.

## 1. Introduction

The basic concept of the Slovak health care system includes mandatory public insurance and a general benefits package for citizens [1].

There is a pluralistic system of health insurance companies, with three health insurance companies operating: the state-owned Všeobecná zdravotná poisťovňa (General Health Insurance Company) and the private companies Dôvera (Trust) and Union, which covered 56.25%, 31.98% and 11.76% of the Slovak population, respectively, in 2021.

Act No. 363/2011 sets the regulations for pharmacoeconomic evaluation and external price referencing in the reimbursement of antibiotics for the primary care sector from health insurance funds [2].

The “maximum retail price” of antibacterials for systemic use may not rise above the average of the three lowest prices of the same antibiotic across the European Union. With the aim of facilitating price erosion after the patent expiry of original antibiotics, the 2018 legislation came out with mandatory discounts for generic antibiotics. According to the legislation, the first off-patent antibiotic entering the Slovak market must achieve a 45% initial price reduction compared to the original antibiotic, the second off-patent antibiotic must achieve an additional 10% price reduction compared to the first and the third off-patent antibiotic must achieve an additional 5% price reduction compared to the second [3].

Market access standards for second and third off-patent antibiotics have developed into rigorous criteria, which could limit some manufacturers from launching generic antibiotics in Slovakia.

The three-step approach to reducing prices has also led to new packaging forms of generic antibiotics. For example, when the package size was changed from 14 to 28 tablets, it was also considered a new generic antibiotic, which complicated the launch of off-patent antibiotics with new package sizes [3]. 

Consumption of antibiotics was considerably higher in Slovakia than in several other EU Member States [4,5], with cephalosporin consumption rising significantly above the average of 30 EU/EEA countries between the third quarter of 2011 and 2017 [6]. A study showed the increase in consumption of quinolone antibacterials between the last quarter of 2001 and the third quarter of 2010 to be significantly greater in Slovakia than the average of 30 EU/EEA countries [7].

The European Centre for Disease Prevention and Control (ECDC) estimated that more than 33,000 patients die each year in the EU/EEA as a consequence of infection with antimicrobial-resistant bacteria [8]. The Organization for Economic Co-operation and Development (OECD) estimated that the financial expenditures associated with AMR could rise to USD 3.5 billion per year if the issue is not addressed [9].

Having valid information on antibiotic consumption is crucial to fighting public health issues related to antimicrobial resistance [10].

A very limited number of new antimicrobial medicines are available; therefore, infection prevention, control measures and the prudent use of existing antimicrobial medicines remain vitally important in the effort to prevent and control antimicrobial resistance [11,12]. Since the current incentive-based approaches are not delivering a sustainable solution for new antimicrobial medicines, The European Commission has proposed a new pharmaceutical strategy for Europe, highlighting that new business models are needed. 

The revision of current pharmaceutical legislation is recommended along with non-legislative measures, improvements in existing regulatory approaches to solve antimicrobial resistance, the promotion of the reasonable use of antimicrobial medicines and the dissemination of information to health care professionals and patients [13].

This paper aims to analyse the consumption of antibacterials for systemic use in the Slovak health care system from 2011 to 2020 and to calculate related quality indicators for outpatient antibiotic use. The high prescription of antibiotics is connected to medical risks and needless financial expenditures; therefore, valid data about the consumption of antibiotics, as discussed in this paper, are needed for appropriate decision making concerning antibiotic policy in Slovakia. 

## 2. Results

We found that the consumption of antibacterials for systemic use in the primary care sector fell by31.29% between 2011 and 2020. In Slovakia, a 47.17% drop can be seen in the consumption of beta-lactam and penicillin antibacterials from 2011 to 2020. It is important to mention the 84.82% increase in the consumption of other antibacterial beta-lactam antibiotics from 2011 to 2018, but there was also a 46.86% decrease for this group of antibiotics from 2018 to 2020. From 2011 to 2020, macrolide, lincosamide and streptogramin use significantly fell by 41.70%. The consumption of quinolone antibacterials declined 47.22% between 2011 and 2020. From 2011 to 2020, tetracycline consumption rose only 16.08% and there was only a 10.81% rise in the consumption of sulphonamides and trimethoprim. In Slovakia, the 133.33% increase in other antibacterial usage from 2011 to 2020 can be seen. 

**Figure 1:**
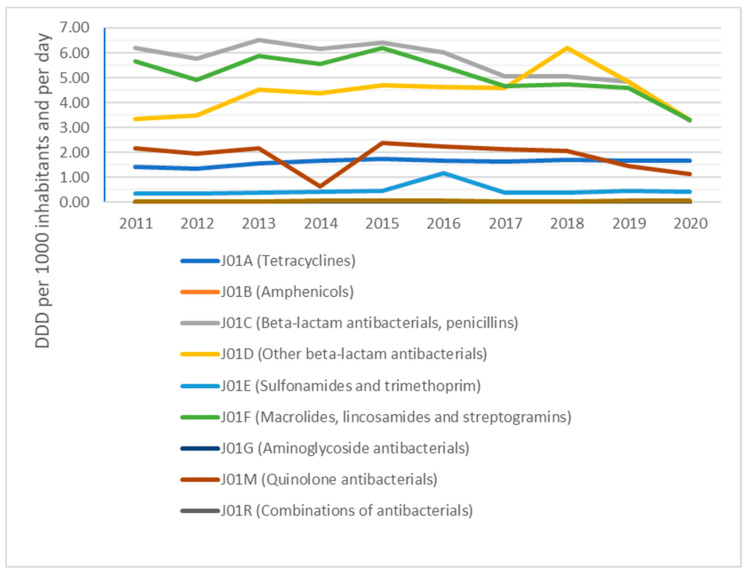
Antibiotic consumption (ATC group J01) in the community.

An overview of antibiotic consumption on the level of the Anatomical Therapeutic Chemical classification system (ATC) in the primary care sector, expressed in DDD per 1000 inhabitants per day, for Slovakia from 2011 to 2020, is available in Figure 1.

The composition of beta-lactam antibacterials, penicillins; other beta-lactam antibacterials; and macrolides, lincosamides and streptogramins was analysed on the level of ATC-4 groups in the community sector.

**Figure 2:**
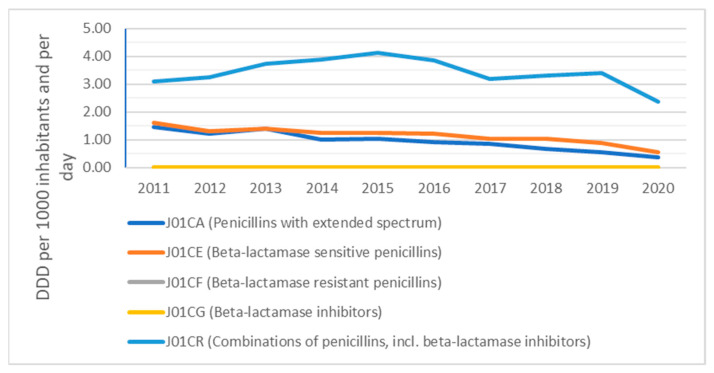
Beta-lactam antibacterials, penicillins (ATC group J01C) in the community.

The overview presented in Figure 2 provides a picture of the consumption of beta-lactam antibacterials, penicillins and their composition on the level of ATC-4 groups in the primary care sector, expressed in DDD per 1000 inhabitants per day, for Slovakia from 2011 to 2020.

The composition of beta-lactam antibacterial penicillins in the community (3.11 DID in 2011 compared to 2.36 DID in 2020) for combinations of penicillins, including beta-lactamase inhibitors; (1.61 DID in 2011 compared to 0.55 DID in 2020) for beta-lactamase-sensitive penicillins; and (1.47 DID in 2011 compared to 0.36 DID in 2020) for the rest of the beta-lactam antibacterial penicillins can be seen.

**Figure 3:**
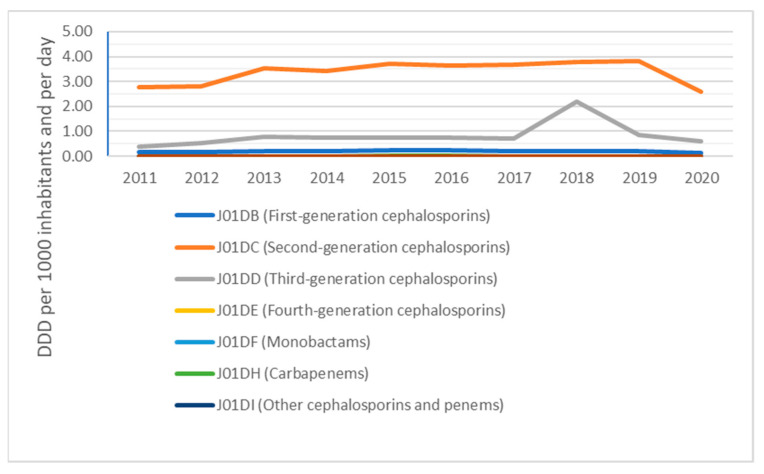
Other beta-lactam antibacterials (ATC group J01D) in the community.

The overview presented in Figure 3 provides a picture of the consumption of other beta-lactam antibacterials and their composition on the level of ATC-4 groups in the primary care sector, expressed in DDD per 1000 inhabitants per day, for Slovakia from 2011 to 2020.

The composition of other beta-lactam antibacterials in the community (2.78 DID in 2011 compared to 2.58 DID in 2020) for second-generation cephalosporins; (0.39 DID in 2011 compared to 0.61 DID in 2020) for third-generation cephalosporins; and (0.18 DID in 2011 compared to 0.12 DID in 2020) for other beta-lactam antibacterials can be seen.

**Figure 4:**
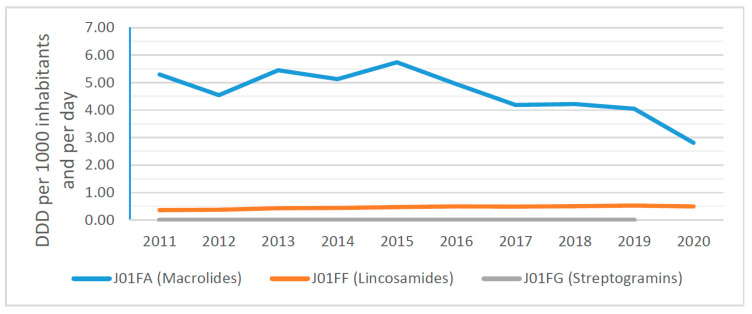
Macrolides, lincosamides and streptogramins (ATC group J01F) in the community.

The overview presented in Figure 4 provides a picture of the consumption of macrolides, lincosamides and streptogramins and their composition on the level of ATC-4 groups in the primary care sector, expressed in DDD per 1000 inhabitants per day, for Slovakia from 2011 to 2020. 

The composition of macrolides, lincosamides and streptogramins (MLS) in the community (5.30 DID in 2011 compared to 2.81 DID in 2020) for macrolides and (0.36 DID in 2011 compared 0.49 DID in 2020) for lincosamides and streptogramins can be seen.

**Figure 5:**
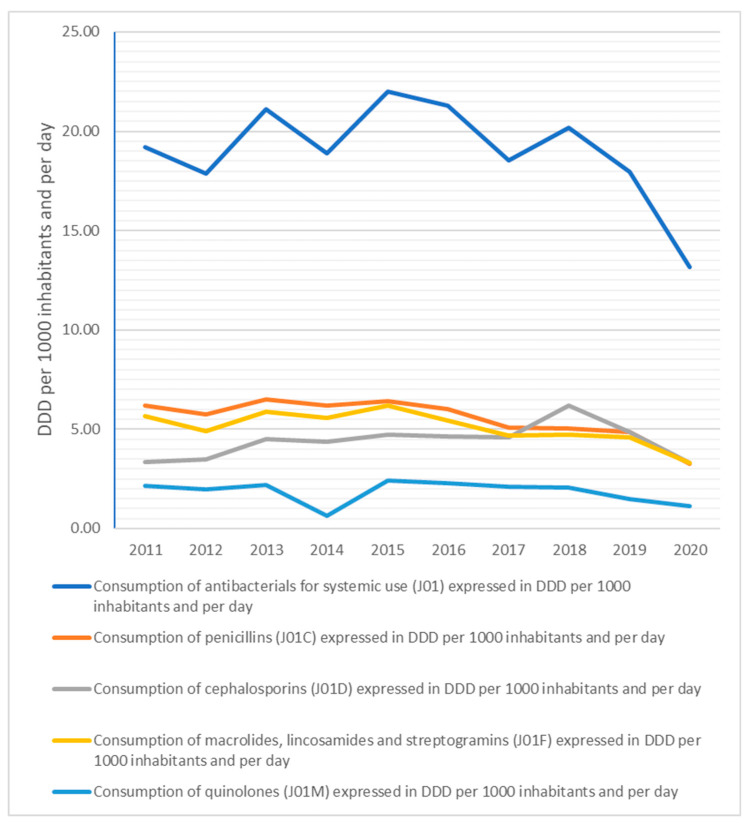
Quality indicators related to the consumption of antibiotics in the community from 2011 to 2020.

**Figure 6:**
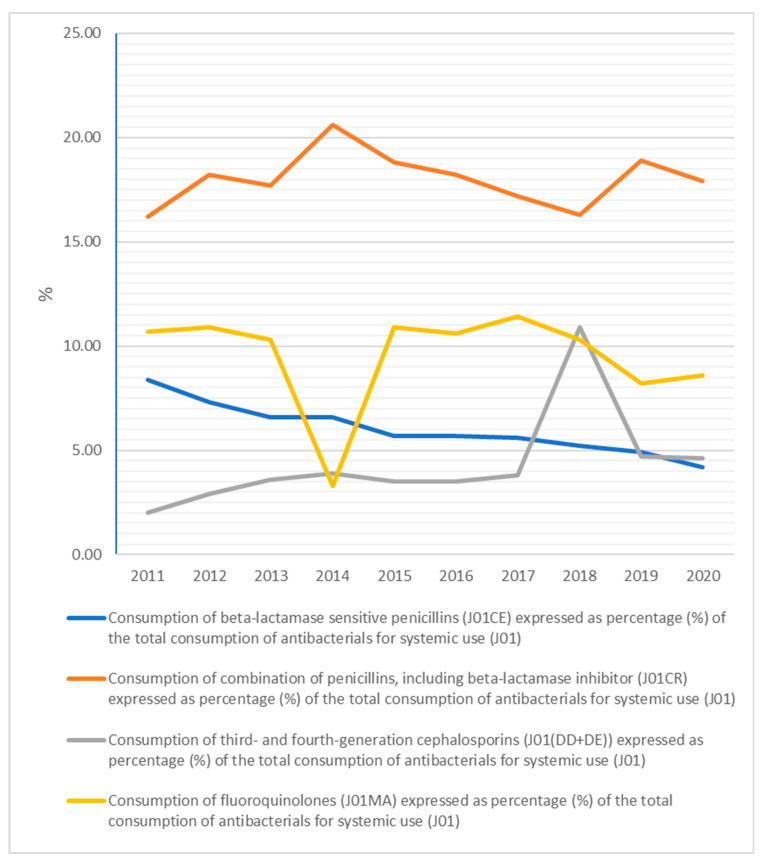
Quality indicators related to the relative consumption of antibiotics in the community from 2011 to 2020.

**Figure 7:**
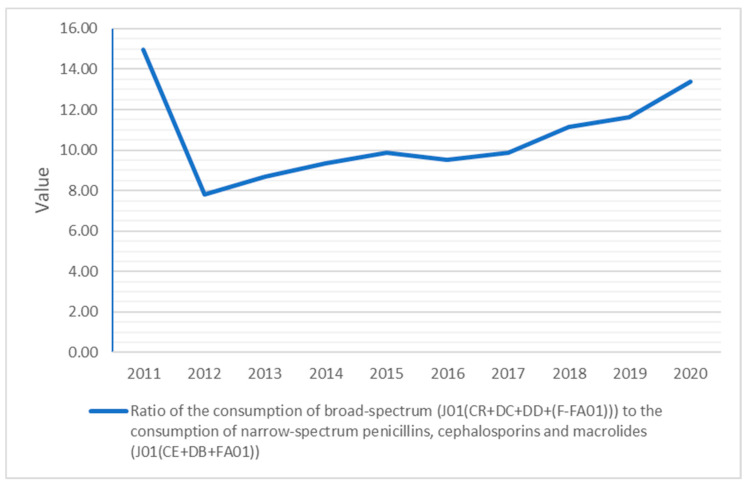
Quality indicators related to the consumption of broad-spectrum and narrow-spectrum antibiotics in the community from 2011 to 2020.

The quality indicators for outpatient antibiotic use, to better describe the Slovak antibiotic prescribing patterns, are calculated in Figure 5, Figure 6 and Figure 7. 

Consumption of antibacterials for systemic use, expressed in DDD per 1000 inhabitants per day, decreased from 19.21 DID in 2011 to 13.17 DID in 2020. Consumption of penicillins, expressed in DDD per 1000 inhabitants per day, decreased from 6.19 DID in 2011 to 3.27 DID in 2020. Consumption of cephalosporins, expressed in DDD per 1000 inhabitants per day, decreased from 3.36 DID in 2011 to 3.30 DID in 2020. Consumption of macrolides, lincosamides and streptogramins, expressed in DDD per 1000 inhabitants per day, decreased from 5.66 DID in 2011 to 3.30 DID in 2020. Consumption of quinolones, expressed in DDD per 1000 inhabitants per day, decreased from 2.16 DID in 2011 to 1.14 DID in 2020.

Consumption of beta-lactamase-sensitive penicillins, expressed as a percentage of the total consumption of antibacterials for systemic use, decreased from 8.4% in 2011 to 4.2% in 2020. Consumption of the combination of penicillins, including beta-lactamase inhibitor, expressed as a percentage of the total consumption of antibacterials for systemic use, increased from 16.2% in 2011 to 17.9% in 2020. Consumption of third- and fourth-generation cephalosporins, expressed as the percentage of the total consumption of antibacterials for systemic use, increased from 2.0% in 2011 to 4.6% in 2020. Consumption of fluoroquinolones, expressed as the percentage of the total consumption of antibacterials for systemic use, decreased from 10.7% in 2011 to 8.6% in 2020.

The ratio of the consumption of broad-spectrum to the consumption of narrow-spectrum penicillins, cephalosporins and macrolides decreased from 14.98 in 2011 to 13.38 in 2020.

## 3. Discussion

High antibiotic consumption and the misuse of antibacterials for systemic use have led to antimicrobial resistance, resulting in a failure of pharmacotherapy, increased financial expenditures for the health care system and an elevated risk of mortality [14]. Addressing the consumption of antibiotics as part of national efforts towards reducing the threat of AMR in Europe plays a key role and needs to be emphasized [15].

Valid data are required to analyse patterns of AMC and to monitor the issue of AMR. Quantitative figures need be considered a starting point for further analysis to better recognize the use of antibiotics in clinical practice [9]. 

We demonstrated that the consumption of antibacterials for systemic use in the primary care sector fell by 31.29% in Slovakia between 2011 and 2020. An international study by Bruyndonckx et al. [16] describes trends in 30 EU/EEA countries, including Slovakia, of antibiotic consumption for the primary care sector and shows a decrease in Slovakia consistent with the results of our study.

In Slovakia, a 47.17% drop can be seen in the consumption of beta-lactam and penicillin antibacterials (J01C) from 2011 to 2020, even though they were the most consumed antibiotic group in the country during the six-year period ending 2017. The study by Bruyndonckx et al. [17] analysing penicillin consumption confirms our conclusions, although the other beta-lactam antibiotic group (J01D) was consumed in Slovakia quite similarly to the beta-lactam penicillin antibacterials (J01C) in terms of DDD during 2019 and 2020. It is important to note the 84.82% increase in the consumption of other antibacterial beta-lactam antibiotics (J01D) in Slovakia from 2011 to 2018, though there was also a 46.86% decrease for this group of antibiotics from 2018 to 2020, with the figure for 2018 consumption of the other antibacterial beta-lactam antibiotics (J01D) being higher than either 2017 or 2019. This was caused by high consumption of third-generation cephalosporin antimicrobials (J01DD), where a rise of 218.84% from 2017 to 2018 and fall of 61.82% from 2018 to 2019 can be seen. The active substance responsible for this increase in consumption is Cefixime (J01DD08), which was consumed in Slovakia at a level of 0.69 DID in 2017, 2.20 DID in 2018 and 0.83 DID in 2019.

A study by Versporten et al. [6] that focuses on cephalosporin consumption likewise detected the shift across the EU/EEA countries towards more broad-spectrum cephalosporin antimicrobial usage in the primary care sector. 

From 2011 to 2020, macrolide, lincosamide and streptogramin (J01F) use signifi-cantly fell by 41.70% in Slovakia. This is consistent with the outcome of an international study by Adriaenssens et al. [18] describing the MLS consumption trend in the primary care sector, which observed a lower consumption of MLS in 2017 than in 2009 in more than half of the EU/EEA countries.

In Slovakia, the consumption of quinolone antibacterials (J01M) declined 47.22% between 2011 and 2020, while an international study by Adriaenssens et al. [7] describes the increase in consumption of quinolone antibacterials (J01M) between the last quarter of 2001 and the third quarter of 2010 as significantly greater in Slovakia than the average of EU/EEA countries. In 2018, the European Medicines Agency (EMA) published a review [19] of serious, disabling and potentially permanent side effects of quinolones and fluoroquinolones involving muscles, tendons, joints and the nervous system. The statement by the EMA had a significant impact on the consumption of quinolone antibacterials (J01M) in Slovakia, as seen in the 46.23% decrease in this group of antibacterials for systematic use between 2017 and 2020.

From 2011 to 2020, tetracycline consumption rose only 16.08% for (J01A) and only 10.81% for sulphonamides and trimethoprim (J01E). The trends are in line with the study published by Versporten et al. [20] about tetracycline, sulphonamide and trime-thoprim consumption between 1997 and 2017, which concluded that it had not changed significantly in EU/EAA countries since 2006. 

This can be seen in the 133.33% increase in other antibacterial usage (J01X) in Slovakia from 2011 to 2020. According to Versporten et al. [20], the average consumption in EU/EEA countries did not significantly change between 1997 and 2017, but there were large variations among the analysed countries. It is worth mentioning that there are no nitrofuran derivatives available among Slovakia’s reimbursable medicines; therefore, the absence of consumption for this antibacterial group in systemic use can be seen and is consistent with the outcome of the published study.

There are different categories of interventions that may support general practitioners (GPs) in reducing the prescribing of antibiotics, namely regulatory, externally administered interventions that GPs can implement individually, supporting GPs’ access to near-patient diagnostic testing [21]. 

From a regulatory point of view, the pricing and reimbursement process, including copayment settings, can be considered as the crucial factor in regard to the impact on antibiotic consumption during the analysed time period in Slovakia. An internal reference pricing system for antibiotics can be seen in Slovakia. Slovakia has implemented a system in which a maximum price is set for a standard daily dose of an antibiotic in each internal reference group (based on ATC) concerning the same active substance and which is administered in the same galenic form. A standard daily dose of a medicine with the particular active substance is reimbursed only at the level of the least expensive antibiotic in the given internal reference group. The difference between the price of antibiotics and the reimbursement levels based on the least expensive antibiotics in a given internal reference group represents the copayment of patients. However, there is also a mandatory copayment for the least expensive antibiotic in a given internal reference group prescribed by general practitioners at the level of 25%. The regulatory impact of copayments on consumption is used to prevent the wasting of antibiotics by patients (moral hazard), which was often seen in the past when all antibiotics were fully covered by the health insurance fund. On the other hand, the copayment does not represent a significant financial burden for patients, which could limit their access to antibiotics. It is important to emphasise that specific groups of antibiotics with restrictive access for prescriptions through physician specialties are fully covered by health insurance funds.

The project Slovak Medical Dialog (S–MedDial), which was established with international cooperation between the General Health Insurance Fund, the Brussels Gentile Insurance Companies Association, the Municipality of Bratislava, the Pharmaceutical Faculty and the Civil Society Health City, was a very important step from an externally administered point of view and has influenced the consumption of antibiotics. The target was to initiate communication between the health insurance fund and general practitioners with the aim of supporting effective antibiotic prescriptions and improving mutual cooperation. The S-MedDial project helped to increase the quality of antibiotic prescriptions through the education of prescribing practitioners. The study of the prescription habits of paediatricians was based on (a) prospective data collection according to treatment registers during a one-month period, and (b) retrospective analysis of health insurance data. Particular regions were studied based on antibiotic resistance trends in cooperation with microbiologists from selected regions [22,23].

From the point of view of supporting GPs’ access to patient diagnostic testing, a study published in 2012 by the Union Health Insurance Fund had a significant impact. The study on the impact of the availability of quick C-reactive protein testing, as a service furnished by medical care providers, and antibiotic treatments involved a population sample of 365,690 health insurance clients from Slovakia over a period of 22 months [24]. Based on the analysis, it was concluded that in the case of the appropriate use of C-reactive protein testing within the monitored sample of the health insurance clients, an increase in the quality of antibiotic prescriptions can be seen. As a result, financial incentives from health insurance funds to use quick C-reactive protein testing by medical care providers were implemented.

In terms of interventions that GPs can implement individually, a significant impact can be seen on projects in which the Trust health insurance company agreed with general practitioners for children and adolescents on an evaluation parameter concerning the consumption of antibiotics in order to prevent their excessive use. This evaluation parameter is also supported financially by the health insurance company.

Through interviews between doctors and representatives of the Trust health insurance company, the following causes of regional differences in antibiotic consumption in outpatient practice were defined.

1. Patient pressure. It is the patients who often demand that doctors prescribe antibiotics, even when there is no reason to do so. A significant number of patients who request a prescription for antibiotics will also receive them.
2. Emergency rooms. Patients often go to emergency rooms for a prescription for antibiotics, even if their GP refuses to prescribe them.
3. Effectiveness of prescriptions and availability of doctors in the region.
4. Economic power of the region. The number of doctor visits is also related to the strength of the economy or the unemployment rate in the region. In the case of economically more developed regions, patients with influenza or other common diseases seek out doctors less often (holidays, housework, “sick days”) than in regions with higher unemployment [25].

It is important to emphasise that Slovakia used rapid antigen tests to target the whole population in an effort to identify SARS-CoV-2 infections in 2020 [26]. A total of 3.6 million people out of an estimated 4 million target adult population were tested with antigenic tests during one weekend in Slovakia to analyse the spread of SARS-CoV-2; however, doubts were cast over the effectiveness of this approach for public health in Slovakia [27,28]. Some types of antibacterials for systemic use, such as azithromycin, have been prescribed for the prevention and treatment of bacterial co-infection and secondary bacterial infections in patients with SARS-CoV-2 [29]. 

Experts have challenged the use of antigen tests, which the WHO declared unsuitable for mass testing unless used alongside PCR tests. There was the possibility of many false-positive and false-negative results, which may have allowed infected people to spread the disease while keeping others under meaningless quarantine. Infectious disease scientists warned that the plan could put people’s health at risk and erode public trust towards testing and all other pandemic containment approaches [22].

Quality indicators for outpatient antibiotic use provide valuable data on the appropriateness of antibiotic consumption [30]. 

The study by Adriaenssens et al. [31] describing the quality appraisal of antibiotic consumption shows that the quality of antibiotic consumption in the community deteriorated between 2009 and 2017, mainly in the countries of Southern and Eastern Europe. A similar situation can also be seen in Slovakia. Even though it has been partially successful in reducing antibiotic consumption, there is significant room for improvement, particularly in the consumption of broad-spectrum antibiotics.

Although the ratio in the consumption of broad-spectrum (J01(CR + DC + DD + (F-FA01))) to narrow-spectrum penicillin, cephalosporin and macrolides (J01(CE + DB + FA01)) fell from 14.98 in 2011 to 7.80 in 2012, it increased to 13.38 in 2020.

Large variations in antibiotic consumption occur between countries, as described by global analyses. Significant differences in the percentage of AMR in key pathogens have been published for both EU/EEA countries and non-EU/EEA European countries [32,33,34]. Relations between the proportions of national antimicrobial consumption and national AMR levels have been described for EU/EEA countries [35]. Comprehensive analyses at the level of individual agents are required to recognize targets in order for national interventions to achieve the responsible use of antibiotics [36,37,38].

A long-term integrated educational programme with financial incentives from health insurance funds for better quality of antibiotic pharmacotherapy shows that prescription habits changed in Slovakia during the analysed time period. It is likely that the concepts of social distancing and restrictions on movement, which were implemented as non-pharmaceutical infection prevention to decrease contact between those who are infected with a disease-causing pathogen and those who are not, are among the reasons for the significant changes in antibiotic consumption in 2020. Other measures implemented during the COVID-19 pandemic in 2020 within the Slovak health care system most likely also played an important role in influencing the consumption of antibacterials for systemic use, and as a result, the related quality indicators for outpatient antibiotic use in 2020, as well.

## 4. Materials and Methods

The data source on the consumption of antibiotics, i.e., antibacterials, for systemic use (ATC group J01) is sales data from the State Institute for Drug Control and the National Health Information Centre. The State Institute for Drug Control (SUKL) represents the Slovak national authority in the area of human pharmacy [39]; there is a fee for database access required and the database is not publicly available [40]. The National Health Information Centre (NCZI) is responsible for delivering data in the field of health statistics [41]. The database of the National Health Information Centre is free of charge and is publicly available. The WHO Anatomical Therapeutic Chemical (ATC) Classification System is used for the allocation of antimicrobials into groups. Defined daily doses per 1000 inhabitants per day is the primary indicator of antibiotic consumption, as determined by the European Commission and the WHO [42].

According to the WHO [43], “The defined daily dose (DDD) is the assumed average maintenance dose per day for a drug used for its main indication in adults.” The study employed the ATC/DDD Index. The role of the ATC/DDD system is to be an instrument for drug-utilization monitoring and research [44,45]. The study focused on the consumption of antibacterials for systemic use in the primary care sector from 2011 to 2020. The main indicator describing antibiotic consumption on ATC-3 groups is the number of DDDs per 1000 inhabitants per day (DID). Selected groups of antibiotics were analysed on the level of ATC-4 groups. The denominator was provided by Eurostat. 

Additionally, we calculated the quality indicators for outpatient antibiotic use, as proposed by Coenen [30], to better describe Slovak antibiotic prescribing patterns in 2020. Data from The European Surveillance System (TESSy), provided by Slovakia and released by the ECDC [46], were used as the additional source for antibiotic consumption.

## 5. Conclusions

Data from the State Institute for Drug Control and the Slovak National Health Information Centre were reported to the European Surveillance of Antimicrobial Consumption Network, which is a Europe-wide network of national surveillance systems providing European reference data on antimicrobial consumption [46]. ESAC-Net provides a crucial platform for establishing the capacity for national monitoring and evaluation. Further cross-national analysis, especially in conjunction with quality measures, can support national activities and interventions to address problems related to prescribing practices concerning antibiotics. Relevant antibiotic consumption data are influential if they are used to update policy developments at the national level.

## Data Availability

The datasets presented in this study can be found in online repositories. The name of the repository can be found at: https://www.ecdc.europa.eu/en/publications-data/european-surveillance-system-tessy (accessed on 31 May 2021).

## Disclaimer

T.T. is responsible for reporting antimicrobial consumption data from Slovakia to the European Surveillance System. “The views and opinions of the authors expressed herein do not necessarily state or reflect those of ECDC. The accuracy of the authors’ statistical analysis and the findings they report are not the responsibility of ECDC. ECDC is not responsible for conclusions or opinions drawn from the data provided. ECDC is not responsible for the correctness of the data and for data management, data merging and data collation after provision of the data. ECDC shall not be held liable for improper or incorrect use of the data”.
